# Body weight and colorectal cancer risk in a cohort of Swedish women: relation varies by age and cancer site

**DOI:** 10.1054/bjoc.2001.1894

**Published:** 2001-08

**Authors:** P, E Giovannucci, L Bergkvist, L Holmberg, A Wolk

**Affiliations:** Department of Medical Epidemiology, Karolinska Institutet, Stockholm, Sweden; Department of Medicine, Channing Laboratory,Brigham and Women’s Hospital, Harvard Medical School, Boston, MA; Department of Surgery and Centre for Clinical Research, Uppsala University, Central Hospital, Västerås, Sweden; Regional Oncologic Centre, Uppsala, Sweden

**Keywords:** colorectal neoplasms, body weight, insulin, menopause, cohort studies

## Abstract

The relation between relative body weight and colorectal cancer among women is unclear. In a large prospective cohort study, we found a positive association only for distal cancers among younger women that became attenuated at older ages. These results support previous reports in which results were stratified by age or colorectal cancer site. © 2001 Cancer Research Campaign http://www.bjcancer.com


*British Journal of Cancer* (2001) **85**(3), 346–349
© 2001 Cancer Research Campaign
doi: 10.1054/ bjoc.2001.1894, available online at http://www.idealibrary.com on IDEAL®

Short Communication

### Body weight and colorectal cancer risk in a cohort of Swedish women: relation varies by age and cancer site

**P Terry,[1] E Giovannucci,[2] L Bergkvist,[3] L Holmberg[4] and A Wolk[1]**

[1]Department of Medical Epidemiology, Karolinska Institutet, Stockholm, Sweden; [2]Channing Laboratory, Department of Medicine, Brigham and Women's Hospital, Harvard Medical School, Boston, MA; [3]Department of Surgery and Centre for Clinical Research, Uppsala University, Central Hospital, Västerås, Sweden; [4]Regional Oncologic Centre, Uppsala, Sweden

**Summary** The relation between relative body weight and colorectal cancer among women is unclear. In a large prospective cohort study, we found a positive association only for distal cancers among younger women that became attenuated at older ages. These results support previous reports in which results were stratified by age or colorectal cancer site. © 2001 Cancer Research Campaign http://www.bjcancer.com

**Keywords**: colorectal neoplasms; body weight; insulin; menopause; cohort studies

Results from epidemiologic studies of the relation between excess body weight and colon cancer risk show consistent positive associations among men (Potter, 1996; Shike, 1996; World Cancer Research Fund, 1997). Weak and inconsistent findings among women, however, suggest that high BMI may not increase their risk of colon or rectal cancer (Potter, 1996; Shike, 1996; World Cancer Research Fund, 1997). The reason for the conflicting results by gender is unknown. Grouping the results of previous studies among women by sub-site and age suggests that BMI might be associated with risk of distal, but not proximal, colon cancer (Graham et al, 1988; West et al, 1989; Dietz et al, 1995; Giovannucci, 1995; Martinez et al, 1997). Moreover, the association has been observed to diminish over increasing strata of age among women (Phillips and Snowdon, 1985; Le Marchand et al, 1992; Slattery et al, 1997; Russo et al, 1998). Such effect modification by age and differences in risk by colorectal cancer sub-site may help to explain the inconsistent findings among women.

The design of our study has been described in detail elsewhere Terry et al, 2001). In brief, from 1987 to 1990 a population-based mammography-screening programme was introduced in 2 counties in central Sweden. The study cohort comprised 61 463 women who had returned questionnaires and were cancer-free at the start of follow-up. These questionnaires included items about age, weight (kg), height (cm), education and diet. Body mass index (BMI) was calculated as weight at baseline (kg)/height ($m^2$). The validity of self-reported BMI was measured in a sample of Swedish individuals as a pearson correlation coefficient between reported and actual BMI ($r$ = 0.893) (Kuskowska-Wolk et al, 1989).

Cases of invasive colon and rectum cancers were identified through matching of the cohort with the computerized Regional Cancer Registers of colon and rectal cancer diagnoses in the 2 counties through December 31, 1998, documented to be 98% complete. Dates of deaths in the cohort were ascertained through the Swedish Death Register and the date of moving out from the study area was obtained through the Swedish Population Register. Cox proportional hazards models were used to estimate hazard rate ratios (RR) with 95% confidence interval (CI). For trend tests, median values for each exposure category of a categorized variable were placed together in the model.

During an average 9.6 years of follow-up of our cohort of 61 463 women (588 270 person-years) we observed 460 incident cases of colorectal cancer (291 colon, 159 rectal, 10 with both colon and rectal cancer). The average age at diagnosis of colon and rectal cancer was 67 and 68 years, respectively. Median body mass index (BMI) varied from 20.8 kg/$m^2$ in the lowest quartile to 29.1 kg/$m^2$ in the highest quartile (Table 1). Age was related positively to BMI, while the percentages receiving post-university education and intake of total energy and alcohol were related inversely with BMI. There were no appreciable differences in median intakes of total fat, total dietary fibre, calcium, vitamin D, vitamin C, folic acid or red meat.

In the entire cohort, age 40–76, BMI was positively, but weakly, associated with risk of colorectal cancer (Table 2). Restricting analyses to women below age 65 at baseline (for comparison with previous studies) revealed stronger associations with colorectal cancer. There was no association among women aged 65 or older at baseline. Further restricting the analysis to women below age 55 years at baseline (the mean age of the cohort), we observed even stronger associations between BMI and colorectal cancer risk, especially for cancers of the distal colon and rectum. There was no association among women aged 55 or older at baseline (formal tests of interaction were not statistically significant). Age-adjusted results (not shown) were similar to multivariate-adjusted results. Using categories of obesity defined by the World Health Organization (WHO, 1998), 'pre-obese' (BMI 25.0–29.9) and 'obese' (BMI 30+), compared to women who were not obese (BMI < 25.0) did not alter our results (not shown). Eliminating cases diagnosed during the first 3 years of follow-up did not alter our results.

We found high body mass (BMI) was associated with risk for distal colon and rectal cancers, but not proximal colon cancer,

*Received 21 March 2001*
*Revised 4 May 2001*
*Accepted 4 May 2001*

*Correspondence to:* P Terry

**Table 1** Baseline characteristics of the study cohort

| Characteristics | Quartiles of body mass index | | | |
|---|---|---|---|---|
| | **1 (*n* = 15 361)** | **2 (*n* = 15 372)** | **3 (*n* = 15 388)** | **4 (*n* = 15 342)** |
| Person years | 146 467 | 146 531 | 147 531 | 147 740 |
| Body mass index (median kg/m$^2$) | 20.8 | 23.1 | 25.2 | 29.1 |
| Age at baseline (median years) | 47 | 51 | 54 | 57 |
| Education (% post-university) | 7.0 | 5.5 | 3.4 | 2.4 |
| Dietary intake (medians)[a] | | | | |
| Energy (kcal d$^{-1}$) | 1367 | 1318 | 1279 | 1242 |
| Fat (g d$^{-1}$) | 45.6 | 45.1 | 45.0 | 45.0 |
| Total dietary fibre (g d$^{-1}$) | 17.0 | 17.1 | 17.1 | 17.0 |
| Vitamin C (mg d$^{-1}$) | 59.4 | 61.3 | 62.2 | 61.8 |
| Foliate (mcg d$^{-1}$) | 185 | 187 | 188 | 189 |
| Calcium (mg d$^{-1}$) | 726 | 740 | 741 | 742 |
| Vitamin D (mcg d$^{-1}$) | 3.2 | 3.3 | 3.4 | 3.5 |
| Red meat (servings wk$^{-1}$) | 4.6 | 4.6 | 4.6 | 4.6 |
| Alcohol (drinks wk$^{-1}$) | 2.8 | 2.5 | 2.0 | 1.4 |

[a]Adjusted to the mean energy score in the cohort of 1350 kcal (except red meat).

**Table 2** Multivariate-adjusted risk ratios according to body mass index for the entire cohort and over strata of age

| Age at baseline | Colorectal[a] | Colon[b] | Proximal colon[b] | Distal colon | Rectum | Distal colon + rectum |
|---|---|---|---|---|---|---|
| All ages (40–76) | *n* = 460 | *n* = 291 | *n* = 118 | *n* = 101 | *n* = 159 | *n* = 260 |
| < 22.0 | 1.0 (referent) | 1.0 (referent) | 1.0 (referent) | 1.0 (referent) | 1.0 (referent) | 1.0 (referent) |
| 22.0–24.2 | 0.97 (0.72–1.30) | 1.05 (0.72–1.51) | 0.94 (0.52–1.69) | 1.06 (0.57–2.00) | 0.92 (0.56–1.54) | 0.99 (0.66–1.45) |
| 24.2–26.7 | 1.09 (0.82–1.43) | 1.09 (0.77–1.56) | 1.06 (0.61–1.83) | 1.27 (0.70–2.29) | 1.14 (0.71–1.83) | 1.19 (0.82–1.72) |
| >26.7 | 1.24 (0.95–1.62) | 1.21 (0.86–1.70) | 1.13 (0.66–1.94) | 1.21 (0.67–2.19) | 1.32 (0.83–2.08) | 1.28 (0.89–1.83) |
| *P* for trend | 0.06 | 0.25 | 0.53 | 0.45 | 0.13 | 0.10 |
| Ages 40–64 | *n* = 267 | *n* = 165 | *n* = 57 | *n* = 62 | *n* = 97 | *n* = 159 |
| < 21.9 | 1.0 (referent) | 1.0 (referent) | 1.0 (referent) | 1.0 (referent) | 1.0 (referent) | 1.0 (referent) |
| 21.9–23.9 | 1.02 (0.70–1.49) | 1.23 (0.77–1.98) | 1.24 (0.58–2.69) | 1.10 (0.49–2.46) | 0.94 (0.51–1.71) | 1.00 (0.62–1.62) |
| 23.9–25.7 | 1.11 (0.77–1.60) | 1.15 (0.72–1.85) | 1.03 (0.47–2.27) | 1.33 (0.62–2.88) | 1.18 (0.67–2.09) | 1.25 (0.79–1.97) |
| >25.7 | 1.39 (0.98–1.97) | 1.58 (1.00–2.49) | 1.51 (0.72–3.18) | 1.54 (0.72–3.30) | 1.30 (0.74–2.30) | 1.43 (0.91–2.24) |
| *P* for trend | 0.04 | 0.06 | 0.34 | 0.21 | 0.25 | 0.07 |
| Ages 40–54 | *n* = 116 | *n* = 71 | *n* = 22 | *n* = 31 | *n* = 43 | *n* = 74 |
| <21.6 | 1.0 (referent) | 1.0 (referent) | 1.0 (referent) | 1.0 (referent) | 1.0 (referent) | 1.0 (referent) |
| 21.6–23.5 | 0.83 (0.47–1.45) | 0.89 (0.45–1.77) | 1.04 (0.33–3.22) | 0.94 (0.32–2.81) | 0.91 (0.33–2.51) | 0.92 (0.44–1.94) |
| 23.5–25.7 | 1.26 (0.75–2.12) | 1.06 (0.54–2.10) | 1.14 (0.36–3.57) | 1.25 (0.43–3.59) | 2.01 (0.84–4.82) | 1.66 (0.85–3.25) |
| >25.7 | 1.74 (1.05–2.87)* | 1.68 (0.89–3.17) | 0.86 (0.24–3.08) | 2.29 (0.87–6.00) | 2.31 (0.96–5.58) | 2.29 (1.20–4.39)* |
| *P* for trend | 0.01 | 0.10 | 0.89 | 0.07 | 0.02 | 0.003 |
| Ages 55–76 | *n* = 344 | *n* = 220 | *n* = 96 | *n* = 70 | *n* = 116 | *n* = 186 |
| < 22.9 | 1.0 (referent) | 1.0 (referent) | 1.0 (referent) | 1.0 (referent) | 1.0 (referent) | 1.0 (referent) |
| 22.9–25.0 | 0.98 (0.69–1.39) | 1.09 (0.70–1.68) | 0.91 (0.46–1.80) | 1.09 (0.51–2.36) | 0.86 (0.48–1.55) | 0.94 (0.59–1.50) |
| 25.0–27.7 | 0.99 (0.71–1.38) | 1.07 (0.71–1.63) | 1.03 (0.55–1.94) | 1.21 (0.59–2.48) | 0.84 (0.48–1.47) | 0.97 (0.62–1.50) |
| >27.7 | 1.07 (0.78–1.47) | 1.08 (0.72–1.62) | 1.16 (0.64–2.13) | 0.89 (0.43–1.87) | 1.00 (0.59–1.69) | 0.96 (0.63–1.47) |
| *P* for trend | 0.60 | 0.78 | 0.47 | 0.73 | 0.89 | 0.92 |

Multivariate models included age in 5 year age groups, education level (less than high school, high school, and university), quartiles of intakes of energy, alcohol, red meat, total fat, folate, vitamin D, vitamin C and calcium. [a]Includes subjects with cancers of the colon, rectum and with cancers of both the colon and the rectum. [b]72 cases of colon cancer were of unspecified location.

among women who were below age 55, the mean age of the cohort at baseline. There was no association among older women.

The weak positive association in the entire cohort is consistent with earlier findings from large cohorts of women with similar age structure. In the large American Cancer Study cohort (Lew and Garfinkel, 1979), with an age structure similar to our cohort, women in the highest category of BMI showed a similar non-significant 22% increased mortality from colorectal cancer compared with women in the lowest BMI category. Likewise, no association was observed for BMI in the Leisure World cohort of retired people (Wu et al, 1987) who were mostly above age 65 at baseline (the exact age range was not reported). Results similar to ours were also observed in a large cohort of Seventh Day Adventists (Phillips and Snowdon, 1985), which found no association among the entire cohort of women with an age structure similar to ours, yet found a clear association among 'younger women' of unspecified age. These studies did not report associations with respect to colon cancer sub-site.

In the large prospective cohort of American nurses (Martinez et al, 1997), where the age range at baseline was 34–59 years, high

*© 2001 Cancer Research Campaign* 

BMI showed a positive, statistically significant association with distal colon cancers, but not proximal colon cancers. This finding is consistent with our results among women below age 65. Three case–control studies examined the association by age and all observed stronger associations among younger compared to older women (Le Marchand et al, 1997; Slattery et al, 1997; Russo et al, 1998).

Examining colon cancer by sub-site, 3 case–control studies found significant associations only with distal colon cancer among women (Dietz et al, 1995; Giovannucci, 1995; Martinez et al, 1997). In addition, 3 studies conducted among men also found significant association only with distal colon cancer (Graham et al, 1988; West et al, 1989; Le Marchand et al, 1992). One of these studies (Le Marchand et al, 1992) found associations with both proximal and distal colon cancers among men and women. In that study, only BMI at age 35, and not BMI 5 years before interview, was significant among women. Finally, another case–control study found stronger associations for cancers of the distal colon among both women and men (Gerhardsson de Verdier et al, 1990). These latter findings were not stratified by age.

We can only speculate about the biologic mechanisms for our observations. Adiposity is related to insulin (Giovannucci et al, 1995), which lowers insulin-like growth factor (IGF) binding protein-1 and may consequently increase levels of free IGF-I (Powell et al, 1991). IGF-I, in turn, has been positively associated with the risk for colorectal cancer in men (Ma et al, 1999) and women (Giovannucci et al, 2000). We do not know of any study that has examined the association between IGF-I and colorectal cancer risk by age or menopause status.

The lack of association between BMI and colorectal cancer risk among older women might be explained in the following way. Hormone replacement therapy (HRT) has been shown to reduce the risk for colorectal cancers (Crandall, 1999) and such benefits have been found to be stronger in the distal colon and rectum (Troisi et al, 1997; Grodstein et al, 1998). Moreover, the observation that HRT may confer greater benefits among lean women (Potter et al, 1996) suggests that oestrogen derived from the adipose tissue may supersede in lowering colorectal cancer risk in older obese women. Moreover, early age at menopause increased the risk of colorectal cancer in a cohort of Dutch women (van Wayenburg, 2000), but only among lean women. Thus, the effects of obesity might be multifactorial in the sense that the deleterious effects may be largely offset among post-menopausal women by the potential benefits through endogenous oestrogens.

We could not adjust our relative risk estimates for physical activity, as this information was not collected at baseline. Energy intake, a rough indicator of physical activity (Willett et al, 1997), was not associated with colorectal cancer in our data, however, and our results were not altered by adjustment for the effects of energy intake. On the other hand, our 67-item food frequency questionnaire may not allow an accurate estimation of energy intake. Therefore, the adjustments we made for total caloric intake may not fully account for between-person variation in energy intake or physical activity, and as a result, some residual effects due to these factors (i.e., confounding) may exist. Although we have no reason to suspect that such confounding would bias our findings only among younger women, this possibility cannot be ruled out. However, several studies have found the effects of physical activity and BMI to be independent (Kune et al, 1990; Giovannucci et al, 1996; White et al, 1996).

In conclusion, our data suggest that high BMI may increase the risk of cancers of the distal colon and rectum only among premenopausal women.

### REFERENCES

Crandall C (1999) Estrogen replacement therapy and colon cancer: a clinical review. *J Womens Health Gend Based Med* **8**: 1155–1166

Dietz A, Newcomb P, Marcus P and Storer B (1995) The association of body size and large bowel cancer risk in Wisconsin (United States) women. *Cancer Causes Control* **6**: 30–36

Gerhardsson de Verdier M, Hagman U, Steineck G, Rieger A and Norell S (1990) Diet, body mass and colorectal cancer: a case-referent study in Stockholm. *Int J Cancer* **46**: 332–338

Giovannucci E (1995) Insulin and colon cancer. *Cancer Causes Control* **6**: 164–179

Giovannucci E, Ascherio A, Rimm E, Colditz G, Stampfer M and Willett W (1995) Physical activity, obesity, and risk for colon cancer and adenoma in men. *Ann Intern Med* **122**: 327–334

Giovannucci E, Colditz GA, Stampfer MJ and Willett WC (1996) Physical activity, obesity, and risk of colorectal adenoma in women (United States). *Cancer Causes Control* **7**: 253–263

Giovannucci E, Pollak MN, Platz EA, Willett WC, Stampfer MJ, Majeed N, Colditz GA, Speizer FE and Hankinson SE (2000) A prospective study of plasma insulin-like growth factor-I and binding protein-3 and risk of colorectal neoplasia in women. *Cancer Epidemiol Biolmarkers Prev* **9**: 345–349

Graham S, Marshall J, Haughey, Mittelman A, Swanson M, Zielezny M, Byers T, Wilkinson G and West D (1988) Dietary epidemiology of cancer of the colon in Western New York. *Am J Epidemiol* **128**: 490–503

Grodstein F, Martinez ME, Platz EA, Giovannucci E, Colditz GA, Kautzky M, Fuchs C and Stampfer MJ (1998) Postmenopausal hormone use and risk for colorectal cancer adenoma. *Ann Intern Med* **128**: 705–712

Kune G, Kune S and Watson LF (1990) Body weight and physical activity as predictors of colorectal cancer risk. *Nutr Cancer* **13**: 9–17

Kuskowska-Wolk A, Karlsson P and Stolt M, Rössner S (1989) The predictive validity of body mass index based on self-reported weight and height. *Int J Obesity* **13**: 441–453

Le Marchand L, Wilkens L and Mi MP (1992) Obesity in youth and middle age and risk of colorectal cancer in men. *Cancer Causes Control* **3**: 349–354

Le Marchand L, Wilens L, Kolonel N, Hankin J and Lyu LC (1997) Associations of sedentary lifestyle, obesity, smoking, alcohol use, and diabetes with the risk of colorectal cancer. *Cancer Research* **57**: 4787–4794

Lew E and Garfinkel L (1979) Variations in mortality by weight among 750,000 men and women. *J Chron Dis* **32**: 563–576

Ma J, Pollak MN, Giovannucci E, Chan JM, Tao Y, Hennekens CH and Stampfer MJ (1999) Prospective study of colorectal cancer risk in men and plasma levels of insulin-like growth factor (IGF)-I and IGF-binding protein-3. *J Natl Cancer Inst* **91**: 620–625

Martinez M, Giovannucci E, Spiegelman D, Hunter D, Willett W and Colditz G (1997) Leisure-time physical activity, body size, and colon cancer in women. *J Natl Cancer Inst* **89**: 948–955

Philips R and Snowdon D (1985) Dietary relationships with fatal colorectal cancer among Seventh-Day Adventists. *J Natl Cancer Inst* **74**: 307–317

Powell DR, Suwanichkul A, Cubbage ML, DePaolis LA, Snuggs MB and Lee PD (1991) Insulin inhibits transcription of the human gene for insulin-like growth factor-binding protein-1. *J Biol Chem* **266**: 18868–18876

Potter J (1996) Nutrition and colorectal cancer. *Cancer Causes Control* **7**: 127–146

Potter JD, Bostick RM, Grandits GA, Fosdick L, Elmer P, Wood J, Grambsch P and Louis TA (1996) Hormone replacement therapy is associated with lower risk of adenomatous polyps of the large bowel: the Minnesota Cancer Prevention Research Unity Case-Control Study. *Cancer Epidemiol Biomarkers Prev* **5**: 779–784

Russo A, Franceschi S, La Vecchia C, Dal Maso L, Montella M, Conti E, Giacosa A, Falcini F and Negri E (1998) Body size and colorectal-cancer risk. *Int J Cancer* **78**: 161–165

Shike M (1996) Body weight and colon cancer. *Am J Clin Nutr* **63**(3 Suppl): 442S–444S

Slattery ML, Potter J, Caan B, Edwards S, Coates A, Ma KN and Berry TD (1997) Energy balance and colon cancer: beyond physical activity. *Cancer Research* **57**: 75–80

 © 2001 Cancer Research Campaign

Terry P, Giovannuci E, Michels KB, Bergkvist L, Hansen H, Holberg L, Wolk A (2001) Fruit, vegetables, dietary fiber, and risk of colorectal cancer. *J Natl Cancer Inst* **91**: 525–533
Troisi R, Schairer C, Chow W, Schatzkin A, Brinton A and Fraumini J (1997) A prospective study of menopausal hormones and risk of colorectal cancer. *Cancer Causes Control* **8**: 130–138
West DW, Slattery ML, Robison LM, Schuman KL, Ford MH, Mahoney AW, Lyon JL and Sorensen AW (1989) Dietary intake and colon cancer: sex-and site-specific associations. *Am J Epidemiol* **130**: 883–894
White E, Jacobs EJ and Daling JR (1996) Physical activity in relation to colon cancer in middle-aged men and women. *Am J Epidemiol* **144**: 42–50
WHO (1998) *Obesity: preventing and managing the global epidemic*. World Health Organization, Geneva, June 3–5, 1997
Willett W and Stampfer M (1986) Total energy intake: implications for epidemiologic analyses. *Am J Epidemiol* **124**: 17–27
Willett WC, Howe GR and Kushi LH (1997) Adjustment for total energy intake in epidemiologic studies. *Am J Clin Nutr* **65**(4 Suppl): 1220S–1228S
World Cancer Research Fund (1997) *Food, nutrition and the prevention of cancer: a global perspective* Washington, D.C.: World Cancer Research Fund/American Institute for Cancer Research
Wu A, Paganini-Hill A, Ross R and Henderson B (1987) Alcohol, physical activity and other risk factors for colorectal cancer: a prospective study. *Br J Cancer* **55**: 687–694

*© 2001 Cancer Research Campaign*

